# Clinical outcomes after SPECT/CT-guided dual medial branch block and radiofrequency ablation for cervical and thoracic facet pain

**DOI:** 10.1016/j.inpm.2026.100819

**Published:** 2026-09-08

**Authors:** Alex Reid, Masilan Sundara, Keith Polston, Lendrum Morrow, James Eubanks, Renee Rosati, Matthew Sherrier, Ameet Nagpal

**Affiliations:** Department of Orthopaedics and Physical Medicine & Rehabilitation, Medical University of South Carolina, Charleston, SC, 29425, United States

**Keywords:** SPECT/CT, Radiofrequency ablation, Medial branch block, Facet joint pain, Cervical spine, Thoracic spine, Imaging concordance, Pain outcomes

## Abstract

**Background:**

Radiofrequency ablation (RFA) is an established treatment for cervical and thoracic facet-mediated pain, but selecting the most appropriate treatment levels remains challenging. Single-photon emission computed tomography/computed tomography (SPECT/CT) has been proposed as an adjunct imaging modality to identify metabolically active facet joints, though its relationship to clinical outcomes is not well defined.

**Objective:**

To describe outcomes following SPECT/CT-guided dual MBB and RFA and to **explore** whether concordance between SPECT/CT findings and RFA target levels was associated with differences in pain and disability.

**Methods:**

This retrospective single-center cohort included patients who underwent SPECT/CT imaging, dual diagnostic medial branch blocks, and subsequent RFA between January 2023 and May 2025. SPECT/CT imaging was ordered at the discretion of the treating physician, and the resulting cohort reflects patients selected for imaging as part of routine clinical care. Pain was assessed using the Numeric Rating Scale (NRS) and disability using the Modified Oswestry Disability Index (MODI). Minimum clinically important difference (MCID) was defined as a ≥34% reduction in NRS and a ≥17% reduction in MODI. An exploratory Fisher's Exact Test with exact odds ratios (OR) and 95% confidence intervals (CI) was used to compare MCID achievement between combined cohorts.

**Results:**

Twenty-two patients met inclusion criteria (cervical n = 16; thoracic n = 6). Subgroups were Concordant (n = 6), Partially Concordant (n = 6), Discordant (n = 3), and No Facet Uptake (n = 7). At first follow-up, 16/18 patients (89%) achieved MCID for NRS and 7/13 (54%) achieved MCID for MODI. The small sample size limited the ability to detect differences between concordance groups (NRS 80% vs 100%, p = 0.48; OR 0.00, 95% CI 0.00–6.62; MODI 62.5% vs 40%, p = 0.59; OR 2.32, 95% CI 0.16–44.94); these results are reported for descriptive purposes.

**Conclusions:**

Clinically meaningful pain reduction following RFA was observed across all concordance categories. The study was not adequately powered to detect an association between SPECT/CT–RFA concordance and clinical outcomes. Observed point estimates and wide confidence intervals are reported for descriptive purposes. These preliminary findings suggest that SPECT/CT may serve as a complementary diagnostic tool within current interventional pain practice. Larger prospective studies with a non-SPECT/CT control arm are needed to further define its role in procedural planning and patient selection.

## 1 Introduction

Neck pain originating from the facet joint, the synovial joint between the superior and inferior articular processes of adjacent vertebrae, accounts for approximately 36% to 67% of chronic neck pain cases [1]. Radiofrequency ablation (RFA) has established efficacy for treating cervical pain refractory to conservative management but lacks a definitive standard for patient selection based on the predicted clinical benefit [[1], [2], [3], [4]].

The most common method of assessing candidacy for RFA uses medial branch blocks, a minimally invasive procedure that involves the injection of local anesthetic around the medial branches of the dorsal roots of spinal nerves [4,5]. Two rounds of medial branch blocks (MBBs), performed on separate occasions (dual MBBs), are commonly used to confirm facetogenic pain and predict the potential benefit of subsequent RFA [4,5]. However, the predictive validity of MBBs can be variable; reported false-positive rates for single diagnostic MBBs range from 27% to 63% in patients with chronic cervical facet pain, compared to false positive rates of 25% to 44% for lumbar facet blocks [1]. Despite this variability, MBBs remain the current gold standard for diagnosing facet-mediated pain and determining RFA candidacy [4,5]. Yet, the criteria used to decide which joints to block are inherently limited, as traditional pain referral maps and physical examination findings show inconsistent correlation with true facet-level localization. Studies in both clinical populations and healthy volunteers have demonstrated considerable variability and overlap in facet pain referral patterns across spinal regions, highlighting the potential for diagnostic error when relying solely on these classical maps [[6], [7], [8], [9]]. Magnetic resonance imaging (MRI) is also commonly incorporated into clinical decision making and may identify facet arthropathy or periarticular edema, suggestive of symptomatic levels. However, degenerative changes are frequently multilevel and relatively symmetric in nature, demonstrating how MRI may also be limited for level selection. Additionally, in regard to the thoracic spine, no universally accepted gold standard exists for selecting thoracic medial branches for radiofrequency denervation, further emphasizing the dependency of procedural decision-making on clinical interpretation and imaging context [10].

Single-photon emission computed tomography (SPECT), a form of radionuclide imaging, allows for anatomical inferences to be made regarding blood flow and metabolic activity related to bone turnover and inflammation [11]. The SPECT/CT imaging modality compensates for SPECT's low spatial resolution of anatomical structures by superimposing CT and SPECT images and provides the combined benefits of SPECT's functional molecular information and CT's high spatial resolution [11]. In the context of augmenting the utility of diagnostic blocks preceding therapeutic injections, Freiermuth et al. reported a diagnostic accuracy of 72% for identifying positive responses to diagnostic MBBs [12].

The purpose of this study is to describe clinical outcomes following dual medial branch blocks (MBBs) and radiofrequency ablation (RFA) for cervical and thoracic facet-mediated pain and to explore whether concordance between SPECT/CT findings and RFA target levels was associated with differences in pain and disability outcomes. There have not been any studies thus far that explore the integration of SPECT/CT with dual MBB and cervical RFA, and it is unclear how SPECT/CT findings relate to clinical outcomes when combined with the current treatment algorithm, which provides the scientific rationale for this study.

## 2 Methods

### 2.1 Subjects

This retrospective chart review was conducted within the Department of Orthopaedics and Physical Medicine & Rehabilitation at the Medical University of South Carolina (MUSC) and received approval from the MUSC Institutional Review Board (Pro #00142929). Patient data was obtained from the electronic medical record for individuals treated by a single physician (AN), between January 2023 and May 2025. The study evaluated clinical and imaging data from patients who underwent SPECT/CT imaging, dual diagnostic MBBs, and RFA for cervical or thoracic facet-mediated pain.

**Fig. 1:**
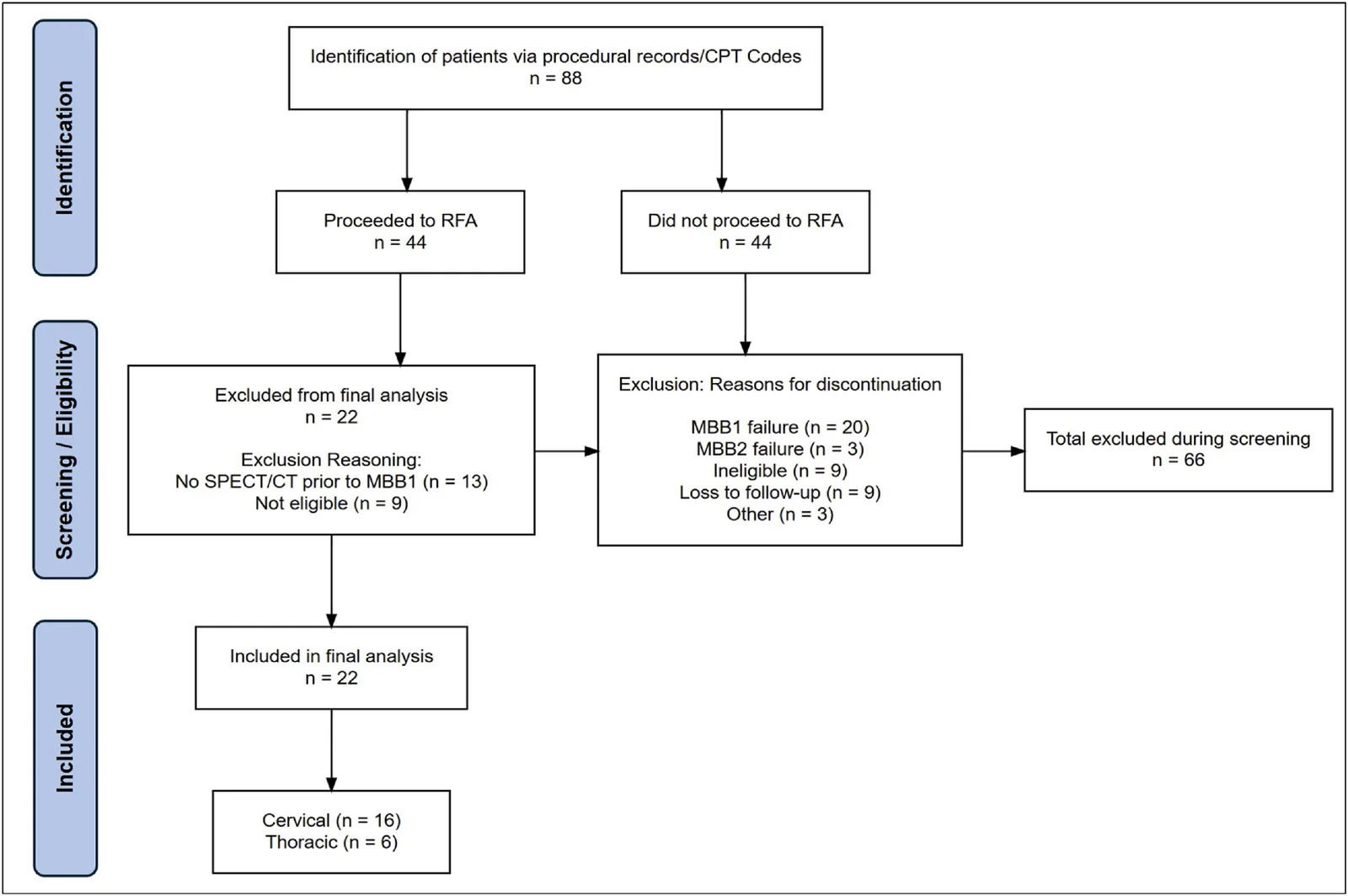
PRISMA-style patient flow diagram illustrating identification, exclusion, and inclusion of patients within the retrospective SPECT/CT-guided MBB and RFA cohort.

Eligible patients were adults who completed the full treatment sequence consisting of SPECT/CT imaging of the cervical or thoracic spine, two diagnostic MBBs performed on separate occasions, and RFA of the corresponding medial branches. Patients were excluded if they did not undergo SPECT/CT imaging prior to the first MBB, did not progress to RFA after dual MBBs, experienced less than 80 percent pain relief following either MBB, lacked documented follow-up outcomes, or were lost to follow-up before completing RFA. Those who discontinued treatment before RFA were not included in the primary outcome analysis but were reviewed to evaluate attrition patterns and potential selection bias within the cohort (Fig. 1).

### 2.2 SPECT/CT examination

As part of the diagnostic evaluation, SPECT/CT imaging was ordered at the discretion of a single physician (AN) when it was expected to provide additional diagnostic clarity and guide subsequent management decisions within the patient's plan of care. For all scans, technetium-99m methylene diphosphonate (99mTc-MDP) was administered intravenously at a typical dose of approximately 21–22 mCi. After an uptake period of about 3 h, planar imaging of the spine was obtained in both anterior and posterior projections, followed by SPECT/CT imaging of the cervical or thoracic region of interest. Low-dose CT imaging was performed to provide anatomical correlation and attenuation adjustment for the SPECT data. SPECT and CT datasets were fused to generate multiplanar reformats for interpretation. Each examination was reviewed by board-certified radiologists at MUSC. Regions of increased radiotracer uptake localized to the facet joints were recorded as positive for facet activity, whereas uptake limited to vertebral bodies, endplates, or other osseous structures was not considered facet specific.

### 2.3 Interventional protocol

Patients who were deemed appropriate candidates based on clinical evaluation and imaging findings, including SPECT/CT results, underwent diagnostic dual MBBs at levels determined by the treating provider. All procedures were performed under fluoroscopic guidance according to institutional standards. After confirmation of needle placement on anteroposterior and lateral views, nonionic contrast (Omnipaque 240 mg/mL) was injected to verify appropriate spread. If no vascular uptake was observed, 0.3–0.5 mL of local anesthetic (0.5% bupivacaine or 2% lidocaine) was administered at each targeted medial branch. Patients demonstrating at least 80% pain relief after both diagnostic blocks were considered candidates for RFA.

RFA procedures were performed under fluoroscopic guidance using sterile technique and local anesthesia. The safety of radiofrequency probe placement was established by utilizing multiplanar fluoroscopy and motor stimulation prior to lesioning. Needles were placed using the pragmatic approach to cervical medial branch radiofrequency lesioning described in the International Pain and Spine Intervention Society Technical Manual and Atlas of Interventional Pain and Spine Procedures [13]. Conventional thermal ablation was performed at each targeted medial branch, and was carried out for 90 s at 80 degrees Celsius. Local anesthetic was injected before and after lesioning. Post-procedure pain assessments and follow-up evaluations were conducted at approximately six weeks (FU1) and three to six months (FU2).

### 2.4 SPECT/CT analysis

Retrospective radiology reports from SPECT/CT imaging were reviewed to document the spinal levels showing increased radiotracer uptake. Corresponding procedural notes from RFA were examined to identify ablated levels. Each case was categorized into one of four predefined groups: (1) Concordant, in which RFA was performed at all and exact levels demonstrating facet uptake on SPECT/CT; (2) Partially Concordant, in which RFA overlapped some but not all uptake levels; (3) Discordant, in which RFA was performed at levels without corresponding uptake; and (4) No Facet Uptake, in which SPECT/CT revealed no facet uptake at any level.

### 2.5 Statistical analysis

Clinical outcomes were extracted from follow-up documentation and patient questionnaires. Pain intensity was assessed using the Numeric Rating Scale (NRS, 0–10), and functional disability was assessed using the Modified Oswestry Disability Index (MODI). Percent change from baseline to each follow-up was calculated for both NRS and MODI. Achievement of the minimum clinically important difference (MCID) was defined as a 34 percent or greater reduction in NRS scores for pain improvement [14] and a 17 percent or greater reduction in MODI scores for disability improvement [15].

Descriptive statistics were used to summarize patient demographics, procedural characteristics, and outcome measures. Continuous variables were reported as means ± standard deviations, and categorical variables as counts and percentages. Differences between cervical and thoracic cohorts were described qualitatively given the limited sample size and were not subjected to formal hypothesis testing.

Given the small sample size and the exploratory nature of this investigation, all statistical comparisons should be interpreted with caution and are reported for descriptive and hypothesis-generating purposes. Individual concordance subgroup outcomes (Table 6, Table 7) are presented descriptively without inferential testing. A total of two Fisher's exact tests were performed at FU1 using combined groups: Concordant and Partially Concordant patients were combined and compared against Discordant and No Facet Uptake patients for both NRS MCID (Table 8) and MODI MCID (Table 9). Exact odds ratios (ORs) with 95% confidence intervals (CIs) were derived from Fisher's Exact Test. Statistical significance was defined as *p* < 0.05. Analyses were conducted using Microsoft Excel and R statistical software (version 4.5.2).

## 3 Results

### 3.1 Cohort results

**Fig. 2:**
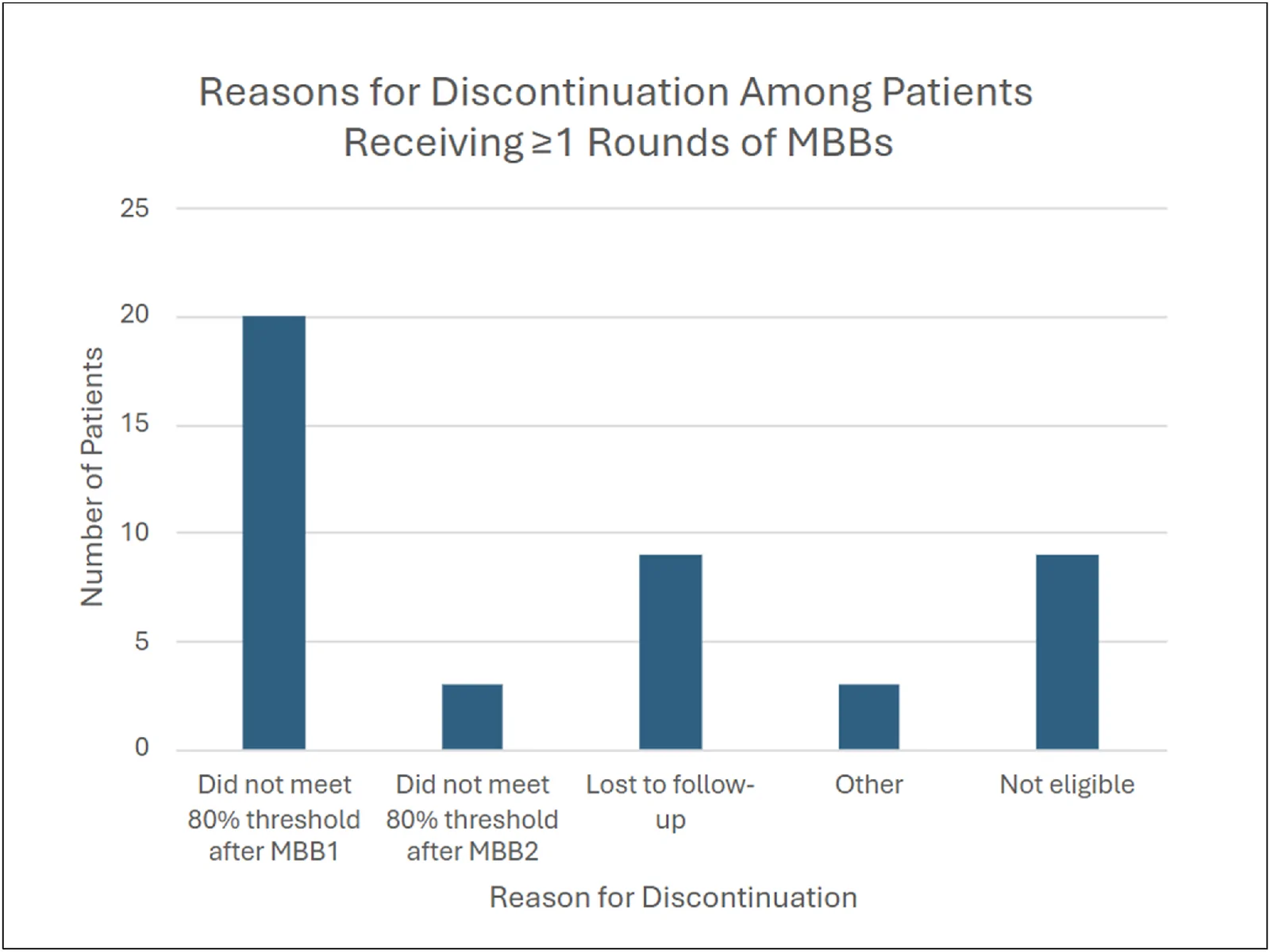
Reasons for discontinuation among patients receiving ≥1 rounds of MBBs.

Between January 2023 and May 2025, 88 patients were identified and associated with the treatment algorithm of SPECT/CT, dual MBBs, and RFA for cervical or thoracic facet-mediated pain. Of these, 44 patients received MBBs but did not proceed to RFA. Reasons for discontinuation included inadequate pain relief following the first MBB (n = 20) or second MBB (n = 3), ineligibility based on study criteria (n = 9), lost to follow-up (n = 9), and other reasons unrelated to treatment response (n = 3) (Fig. 2).

Among the nine patients lost to follow-up, six discontinued after the first diagnostic MBB (n = 6), two after the second MBB (n = 2), and one after SPECT/CT but before any interventional procedure (n = 1). These patients were excluded from the primary outcome analysis but were reviewed descriptively to assess attrition-related bias and factors contributing to incomplete treatment progression:

• Reasons for discontinuation varied across this specific subset of patients (n = 9) who were lost to follow-up. After the first MBB, one patient experienced an intercurrent illness requiring antibiotics and was unable to return for follow-up, one had repeated scheduling cancellations and no-shows, one did not adequately assess pain response following MBB1 and subsequently canceled further care, one reported complete pain resolution after MBB1 and did not require additional intervention, one canceled the planned follow-up visit without rescheduling, and one was unable to proceed due to insurance authorization barriers. Among those lost after the second MBB (n = 2), one demonstrated 80% relief but expressed anxiety regarding proceeding to radiofrequency ablation, while another achieved 90% relief of the initial pain but developed new lower cervical myofascial pain that altered subsequent management. The remaining patient was lost after SPECT/CT imaging but before any interventional procedure (n = 1) when imaging findings prompted a change in clinical management toward alternative conservative/injection-based treatment.

Despite the previously aforementioned reasons, the majority of patients in this subset demonstrated meaningful pain relief (≥80% pain relief) at the time of discontinuation. When response was stratified according to the most advanced diagnostic block with post-procedural pain assessment, meaningful pain relief was seen in 5 patients following MBB1 (55.6%), and in 2 patients following MBB2 (22.2%). Pain response could not be evaluated in 2 patients who discontinued treatment prior to post-procedural assessment (Table 1).

**Table 1:** Pain relief threshold met among patients lost to follow-up prior to radiofrequency ablation (n = 9).

Furthest diagnostic stage at which pain relief threshold was met (≥80% pain relief)	Number of patients (n)	Percentage (%)
After MBB1	5	55.60%
After MBB2	2	22.20%
Pain relief threshold not evaluable	2	22.20%

Total	9	

Of the remaining 44 patients who underwent RFA, a total of 22 patients met inclusion criteria and were included in the final analysis. These patients completed the full sequence of SPECT/CT imaging, dual MBBs, and RFA. The cohort was divided into cervical (n = 16) and thoracic (n = 6) subgroups based on the region of treatment. (Table 2).

**Table 2:** Baseline demographic characteristics of patients included in the final analysis.

Group	N	Mean Age ± SD	Male	Female
Cervical	16	68.4 ± 15.6	8	8
Thoracic	6	49.2 ± 20	2	4

Total	22	63.2 ± 18.6	10	12

**Table 3:** Procedural and follow-up characteristics of patients included in the final analysis.

Group	Completed RFA (n)	SPECT/CT to RFA (days, mean ± SD)	Completed F/U 1 (n)	Days to F/U 1 (mean ± SD)	Completed F/U 2 (n)	Days to F/U 2 (mean ± SD)
Cervical	16	117 ± 62.9	15	53.7 ± 22.8	10	174 ± 54.4
Thoracic	6	94.8 ± 27.1	5	55 ± 12.6	3	152 ± 52.0
Total	22	111 ± 55.7	20	54 ± 20.5	13	169 ± 52.5

**Table 4:** Percent change in NRS scores from baseline to follow-up 1 and 2.

Group	Baseline → FU1	Baseline → FU2
Cervical	−57.4% ± 51.7% (n = 14)	−66.7% ± 33.2% (n = 9)
Thoracic	−13.2% ± 85.5% (n = 4)	−50.0% ± 70.7% (n = 2)
Total	−47.6% ± 60.7% (n = 18)	−63.6% ± 37.8% (n = 11)

**Table 5:** MODI scores by group and timepoint.

Group	Baseline	Follow-Up 1	Follow-Up 2
Cervical	33.9 ± 17.5 (n = 16)	24.7 ± 22.3 (n = 12)	43.4 ± 20.7 (n = 7)
Thoracic	32.0 ± 15.7 (n = 5)	41.0 ± 1.4 (n = 2)	30.0 ± 2.8 (n = 2)
Total	33.4 ± 16.7 (n = 21)	27.0 ± 21.4 (n = 14)	40.4 ± 18.9 (n = 9)

**Note.** One thoracic patient did not have baseline MODI data available, resulting in n = 21 for this analysis.

The mean interval between SPECT/CT imaging and subsequent RFA was 111 ± 55.7 days across the total cohort, with 117 ± 62.9 days for cervical patients and 94.8 ± 27.1 days for thoracic patients. The average duration from RFA to the first follow-up visit (FU1) was approximately 54 ± 20.5 days, while the second follow-up (FU2) occurred at a mean of 169 ± 52.5 days (Table 3). These intervals reflect typical scheduling patterns in the clinical workflow and indicate that most patients were assessed at approximately 6-8 weeks and 5-6 months post-procedure, respectively.

Patients were also categorized into four groups based on the relationship between SPECT/CT findings and RFA levels: Concordant (n = 6), Partially Concordant (n = 6), Discordant (n = 3), and No Facet Uptake (n = 7). Among cervical cases, 6 were classified as Concordant and 5 as Partially Concordant, while among thoracic cases, 0 were Concordant, and 1 was Partially Concordant.

### 3.2 Pain and disability related outcomes

Among the 22 patients who completed the full SPECT/CT–MBB–RFA treatment algorithm, both cervical and thoracic groups demonstrated clinically meaningful reductions in pain intensity at follow-up visits, and percentage improvement in NRS pain scores from baseline to each follow-up is summarized in Table 4. Across the total cohort, the mean percent change in NRS pain scores was −47.6% ± 60.7% from baseline to the first follow-up (FU1, n = 18) and −63.6% ± 37.8% from baseline to the second follow-up (FU2, n = 11). Within the cervical subgroup, pain scores improved by −57.4% ± 51.7% at FU1 and –66.7% ± 33.2% at FU2. The thoracic subgroup demonstrated a comparatively smaller but still notable improvement, with −13.2% ± 85.5% at FU1 and –50.0% ± 70.7% at FU2.

Using a MCID threshold of −34% change in NRS, 12 of 14 cervical patients (86%) and all 4 thoracic patients (100%) achieved MCID at FU1. Sustained improvement was observed at FU2, with 8 of 9 cervical (89%) and 1 of 2 thoracic (50%) patients continuing to meet MCID. Overall, 16 of 18 patients (89%) achieved MCID for pain at FU1, and 9 of 11 (82%) maintained this improvement at FU2. These findings suggest that, on average, both cervical and thoracic subgroups experienced clinically meaningful and sustained pain reduction through follow-up, with the magnitude of improvement being greatest in the cervical cohort.

In contrast, changes in functional disability, as measured by the MODI, were more variable between groups and over time. Baseline MODI scores were available for 21 of 22 patients. Across the total cohort, mean MODI scores improved from 33.4 ± 16.7 at baseline to 27.0 ± 21.4 at FU1 (n = 14), followed by an increase to 40.4 ± 18.9 at FU2 (n = 9). Within subgroup analyses, cervical patients demonstrated a modest reduction in disability at FU1 (33.9 ± 17.5 to 24.7 ± 22.3, n = 12) that was not maintained at FU2 (43.4 ± 20.7, n = 7). Thoracic patients showed greater variability, with mean scores increasing from 32.0 ± 15.7 at baseline to 41.0 ± 1.4 at FU1 (n = 2) and improving to 30.0 ± 2.8 at FU2 (n = 2).

Using a MCID threshold of −17% change in MODI, 7 of 13 patients (54%) achieved MCID at FU1, while only 1 of 10 patients (10%) met this criterion at FU2. These findings suggest that improvements in disability were inconsistent between groups and diminished over time, in contrast to the more stable and sustained reductions in reported pain (Table 5).

### 3.3 Descriptive concordance subgroup outcomes

The 22 patients who completed the full SPECT/CT–MBB–RFA treatment sequence were stratified across four concordance-based subgroups: Concordant (n = 6, 27%), Partially Concordant (n = 6, 27%), Discordant (n = 3, 14%), and No Facet Uptake (n = 7, 32%).

Achievement of the NRS MCID (−34%) was high across all subgroups. At the first follow-up (FU1), 5 of 5 Concordant, 3 of 5 Partially Concordant, 3 of 3 Discordant, and 5 of 5 No Facet Uptake patients achieved MCID. Sustained improvement was observed at the second follow-up (FU2), with 2 of 3 Concordant, 2 of 2 Partially Concordant, 3 of 3 Discordant, and 2 of 3 No Facet Uptake patients continuing to meet MCID. Overall, 16 of 18 patients (89%) achieved MCID at FU1, and 9 of 11 (82%) maintained improvement at FU2 (Table 6). These subgroup-level proportions are presented for descriptive purposes only given the small cell sizes. Pain reduction following RFA appeared consistent across concordance classifications, with no distinct pattern suggesting superior outcomes in patients whose RFA targets aligned exactly with SPECT/CT uptake levels.

**Table 6:** Proportion of patients achieving MCID in NRS scores at Follow-up 1 (FU1) and Follow-up 2 (FU2), stratified by SPECT/CT uptake subgroup.

Subgroup	NRS MCID Met at FU1	NRS MCID Met at FU2
Concordant	5/5	2/3
Partially Concordant	3/5	2/2
Discordant	3/3	3/3
No Facet Uptake	5/5	2/3

Total	16/18 (89%)	9/11 (82%)

**Note.** Data is presented descriptively; no inferential testing was performed across individual subgroups.

**Table 7:** Proportion of patients achieving MCID in MODI scores at Follow-up 1 (FU1) and Follow-up 2 (FU2), stratified by SPECT/CT uptake subgroup.

Subgroup	MODI MCID Met at FU1	MODI MCID Met at FU2
Concordant	2/4	1/4
Partially Concordant	3/4	0/1
Discordant	0/2	0/2
No Facet Uptake	2/3	0/3

Total	7/13 (54%)	1/10 (10%)

**Note.** Data is presented descriptively; no inferential testing was performed across individual subgroups.

**Table 8:** Fisher's exact test results comparing combined concordance groups for MCID achievement (NRS, FU1).

Group	MCID Achieved (n)	MCID Not Achieved (n)	Total (n)
Concordant + Partially Concordant	8	2	10
Discordant + No Facet Uptake	8	0	8
Total	16	2	18

P-value	0.48		

**Note.** Fisher's Exact Test comparing MCID achievement (≥34% reduction in NRS) between combined concordance groups at FU1. MCID was achieved by 80% of Concordant + Partially Concordant patients vs 100% of Discordant + No Facet Uptake patients (p = 0.48, two-tailed; OR 0.0, 95% CI 0.00-6.62). Wide confidence intervals reflect the limited sample size and should be interpreted with caution.

**Table 9:** Fisher's exact test results comparing combined concordance groups for MCID achievement (MODI, FU1).

Group	MCID Achieved (n)	MCID Not Achieved (n)	Total (n)
Concordant + Partially Concordant	5	3	8
Discordant + No Facet Uptake	2	3	5
Total	7	6	13

P-value	0.59		

**Note.** Fisher's Exact Test comparing MCID achievement (≥17% MODI reduction) between combined concordance **groups** at FU1.MCID was achieved by 62.5% of Concordant + Partially Concordant patients vs 40% of Discordant + No Facet Uptake patients (p = 0.59, two-tailed; OR 2.32, 95% CI 0.16-44.94). Wide confidence intervals reflect the limited sample size and should be interpreted with caution.

In contrast, disability outcomes measured by the MODI were more variable. At FU1, MCID (−17%) was achieved by 2 of 4 Concordant, 3 of 4 Partially Concordant, 0 of 2 Discordant, and 2 of 3 No Facet Uptake patients. By FU2, only 1 Concordant patient continued to meet MCID criteria. Overall, improvements in disability were less stable and not sustained across subgroups (Table 7). These subgroup-level proportions are presented for descriptive purposes only given the small cell sizes. Functional outcomes were more heterogeneous in nature and may be associated with factors beyond imaging concordance or procedural alignment.

### 3.4 Combined concordance analysis for NRS at follow-up 1

Concordant and Partially Concordant groups were combined and compared with Discordant and No Facet Uptake groups for MCID achievement on the NRS at the first follow-up. MCID for NRS was achieved by 8 of 10 (80%) patients in the Concordant + Partially Concordant group and 8 of 8 (100%) in the Discordant + No Facet Uptake group. This exploratory analysis was not adequately powered to detect a difference between the combined groups (p = 0.48). The estimated odds ratio was 0.0 (95% CI 0.00-6.62), reflecting the absence of failures in the Discordant + No Facet Uptake group. Given the limited sample size, these results should be interpreted as hypothesis-generating rather than confirmatory (Table 8).

### 3.5 Combined concordance analysis for MODI at follow-up 1

Using MODI outcomes at FU1, 5 of 8 (62.5%) patients in the Concordant + Partially Concordant group and 2 of 5 (40%) in the Discordant + No Facet Uptake group achieved MCID (−17%). This exploratory analysis was not adequately powered to detect a difference between groups (p = 0.59). The estimated odds ratio was 2.32 (95% CI 0.16-44.94). As with the NRS analysis, these findings are presented for descriptive purposes and are limited by the sample size (Table 9).

### 3.6 Prior interventions

Among the 22 patients included in the analysis, 4 patients (18%) had a history of significant prior spinal intervention with respect to prior MBB, RFA, or surgical hardware. Each case was reviewed individually to identify overlap between the anatomical levels of prior procedures and those involved in the current SPECT/CT–guided treatment algorithm.

• One patient previously underwent anterior cervical discectomy and fusion (ACDF) at C2–3 and C4–5, with a distant RFA reported several years prior. In the current algorithm, SPECT/CT demonstrated focal uptake at the C2–3 facet level, corresponding to one of the fused levels, while RFA was performed at C5–6. Although categorized as Discordant based on SPECT/CT–RFA alignment, this case exhibited overlap between prior surgical levels and current SPECT/CT activity, suggesting possible residual or adjacent segment degeneration.
• One patient had a history of C5–7 ACDF. SPECT/CT demonstrated a classification of No Facet Uptake. Additionally, MBBs and subsequent RFA targeted the C7–T1, T1–2, and T2–3 facet joints. These levels are caudal to the prior surgical levels, and this case represents one in which overlap was absent.
• One patient had a prior posterior spinal fusion extending from T1 to T7. SPECT/CT revealed uptake at the T4–5, T5–6, T6–7, and T7–8 facet levels while RFA was performed at T3–4, T4–5, and T5–6. This case demonstrated substantial anatomical overlap between fused segments and both SPECT/CT uptake and RFA target levels and was classified as Partially Concordant.
• One patient previously underwent MBB and RFA at C4–5 and C5–6. In the current sequence, SPECT/CT uptake was observed at the C2–3, C4–5, and C7–T1 facet levels, with RFA performed at C2–3 and C4–5, indicating partial overlap with prior procedural sites and a Partially Concordant classification.

Overall, 3 of 4 cases (75%) demonstrated at least partial anatomical overlap between prior interventions and either SPECT/CT uptake or current RFA targets. These findings suggest that previous instrumentation or ablation may influence subsequent imaging patterns or procedural targeting in select cases, although the limited number of affected patients precludes definitive conclusions.

## 4 Discussion

This retrospective review described clinical outcomes of patients undergoing SPECT/CT–guided dual MBB and RFA for cervical and thoracic facet-mediated pain and explored whether concordance between SPECT/CT uptake and RFA target levels was associated with differences in post-procedural pain and disability outcomes. A secondary exploratory analysis using Fisher's Exact Test further examined whether outcomes differed between imaging-supported and clinically driven cases by combining groups to potentially improve statistical power.

### 4.1 Clinical interpretation of discordance

While SPECT/CT imaging provides valuable insight into potential sites of increased osseous turnover and facet inflammation, clinical decision-making for RFA is inherently multifactorial. Discordance between imaging uptake and ablation levels may reflect thoughtful integration of imaging findings with patient history, physical examination, and procedural response. In this cohort, several patients categorized as Discordant achieved meaningful reductions in pain, highlighting that physician-guided targeting may effectively capture symptomatic levels even when not visualized on imaging. These results reinforce that SPECT/CT serves best as a complementary diagnostic tool, enhancing but not replacing clinical judgment.

### 4.2 Challenges in uptake interpretation

Interpretation of radiotracer activity is complex, particularly when uptake localizes to the vertebral body, uncovertebral joints, endplates, or other osseous structures rather than the facet joints themselves. This pattern can occur secondary to degenerative changes or post-surgical alterations, which may confound the association between uptake and facetogenic pain. Differentiating true facet uptake from adjacent non-facet activity is therefore essential to avoid misdirected interventions and emphasizes the need for standardized interpretive criteria across institutions. Alternatively, increased uptake within the vertebral body, uncovertebral joints, or intervertebral discs may contribute to altered load distribution and mechanical stress on adjacent facet joints, potentially serving as an indirect source of facet-mediated pain. This possibility highlights the need for future studies to investigate biomechanical relationships between regional uptake patterns and pain generation.

Another consideration stems from the qualitative distinction between major and minor foci of SPECT/CT uptake. Variability in tracer intensity may reflect differences in the degree or activity of facet joint arthropathy; however, the clinical implications of these differences remain unclear. Visual and quantitative standardized scoring systems have been adopted for SPECT in other applications such as the Perugini scale and Heart-to-contralateral lung ratio in cases of cardiac amyloidosis [16]. Similarly, quantitative SPECT/CT with standardized uptake value (SUV) measurement is increasingly used as an index to differentiate benign from malignant bone lesions with several studies highlighting its utility in the case of characterizing bone metastases [[17], [18], [19]]. However, there is currently no standardized method for facet joint uptake interpretation. This highlights an important area for future investigation, as standardized uptake grading/thresholds for facet joint interpretation may prove beneficial in determining whether the magnitude of uptake correlates with symptom severity or procedural outcomes.

### 4.3 Fisher's analysis: clinical and methodologic implications

Because subgroup sample sizes were small and unevenly distributed, two Fisher's Exact tests were performed to address limited statistical power and to examine a broader clinical question: Is RFA in imaging-supported versus clinically driven cases associated with different outcomes?

This exploratory analysis was not adequately powered to detect an association between SPECT/CT-RFA concordance and clinical outcomes. Observed point estimates and wide confidence intervals are reported for descriptive purposes. The uniformly high rate of pain improvement across all groups suggests that favorable outcomes may be achievable even when imaging and procedural levels are not perfectly aligned, supporting the continued role of individualized clinical decision-making.

From a methodological standpoint, combining groups aimed to mitigate instability caused by small cell counts (several subgroups contained fewer than five cases) and provided a more balanced comparison reflective of real-world reasoning. This consolidation also represented a conceptual distinction between a more imaging-guided cohort (Concordant + Partially Concordant) and a more clinically driven cohort (Discordant + No Facet Uptake). Similar outcomes between these groups underscore the multifaceted nature of facet-mediated pain and suggest that both approaches may have the capacity to yield meaningful improvement when integrated within this framework.

Given the small sample size, the absence of statistical significance should be interpreted cautiously due to the risk of Type II error. These findings should not be taken as a lack of clinical relevance, but as preliminary observations that lay the foundation for adequately powered prospective studies to determine whether integrating SPECT/CT imaging with clinical judgment can optimize patient selection and procedural outcomes.

### 4.4 Influence of prior instrumentation

A minority of patients (4 of 22) had prior spinal surgery or previous RFA, which may alter both SPECT/CT appearance and procedural strategy. Fusion or prior ablation can modify facet joint biomechanics, often producing increased radiotracer activity at adjacent, compensatory levels, a phenomenon consistent with adjacent segment degeneration. Overlap between fusion levels and current SPECT/CT uptake in several cases supports this mechanism and illustrates how prior instrumentation can complicate interpretation and procedural targeting.

### 4.5 Strengths & limitations

This study has several notable strengths. First, the study reflects real-world outcomes from patients undergoing SPECT/CT guided MBB and RFA within a current clinical treatment algorithm. Ultimately, this highlights the external validity and clinical relevance of this study's central aims and findings. Second, the study design enabled evaluation and insight into associations between the SPECT/CT guided treatment algorithm and patient outcomes across multiple concordance categories. Third, the findings offer an initial foundation that can inform the design and development of future prospective trials evaluating the role of SPECT/CT in facet-mediated pain management.

With respect to limitations, there are several that must be acknowledged. Primarily, a significant limitation is the retrospective cohort design. Due to this, causal inferences cannot be drawn.Also, the absence of a control arm receiving MBB and RFA without SPECT/CT precludes causal inference regarding the added value of SPECT/CT imaging. The observed associations should therefore be considered exploratory in nature. Additionally, findings from this study are vulnerable to confounding by indication, as only patients who received SPECT/CT were included. Patients selected for SPECT/CT may have had more complex presentations or greater diagnostic uncertainty, which could influence outcomes. Beyond this, inherent challenges stem from the realms of clinician decision-making bias, selection bias, and regression to the mean. These biases may arise from variability in physician-directed level selection, differences between patients who pursued SPECT/CT and those who did not, and fluctuating symptoms throughout the time interval between imaging, MBB, and RFA. Additionally, because SPECT/CT interpretation was performed by radiologists at a single institution, inter-rater reliability could not be assessed; future multicenter studies would be needed to evaluate reproducibility across readers and sites. Lastly, several unmeasured clinical variables were uncontrolled. These variables include concurrent treatments, the disposition of patients willing to receive SPECT/CT versus those who were not, and other psychosocial or biological factors, which may have influenced treatment response. Ultimately, these limitations provide reasoning for larger standardized prospective trials with predefined criteria and controlled confounders.

### 4.6 Future directions

Overall, the findings of this study suggest that discordance between imaging and procedural levels may reflect individualized clinical decision-making. SPECT/CT offers valuable adjunctive information but cannot substitute for a comprehensive clinical assessment integrating patient history, examination, and procedural feedback. The secondary analysis further supports that both imaging-supported and clinically driven approaches may achieve favorable outcomes within a dual-MBB RFA framework.

Future prospective studies should include a non-SPECT/CT control arm, standardized SPECT/CT interpretation criteria, and adequate sample sizes to determine whether imaging integration can enhance diagnostic specificity, improve procedural efficiency, or predict long-term response.

## Sources of funding

This research did not receive any specific grant from funding agencies in the public, commercial, or not-for-profit sectors.

## Disclosure of conflicts of interest

The authors do not have any conflicts of interest to disclose.
